# Tartar Emetic, Quinine and Morphine in Pneumonia of Miasmatic Districts

**Published:** 1850-01

**Authors:** E. C. Banks

**Affiliations:** Lawrenceville, Illinois


### ARTICLE VII.

*Tartar Emetic, Quinine and Morphine, in Pneumonia of Miasmatic Districts.*—By E. C. Banks, M.D., of Lawrenceville, Illinois.

Of course, I do not mean to say, but what Mercury, in some form, must precede, accompany, or follow the administration of these medicines, as a general rule, to a greater or less extent. Indeed, hardly a case occurs, in the treatment of which, Mercury could advantageously be dispensed with. In thus openly advocating a course of practice not generally adopted by the faculty, nor even recommended by the learned and scientific of the profession, I lay myself open to criticism; yet I am glad to find one man, (Dr. Howland,) who openly advocates a similar course of treatment. I have long been tempted to give the result of my experience in the treatment of this Protean Malady, but have still held on, in hopes to mature my opinions on the subject, and if possible, come across the writings of some one, who corresponded in opinion with myself on this interesting subject. I have at last found that man, in the person of Dr. Howland, of Ottawa, Ills., writing through the columns of the "North-Western Med. and Surg. Journal" for April and May, 1849. I have never seen any thing like this treatment advised in any of the scientific works of the day, nor even in any of the periodicals, until I saw it in your valuable Journal.

The great mortality attending the accustomed mode of treatment, and the one sanctioned by the learned in all the medical works of the day, necessarily caused me to cast about to find some more successful mode of treating the different forms of Lung Fever, prevailing so extensively throughout this section during the months of February, March, April, and sometimes May, I found Quinine would, as a general thing, cut short an attack of Pneumonitis, given after the patient's system had become prepared by general or local blood-letting, and mild purgatives of either Calomel and Rhubarb, Calomel and Soda, or Calomel and Jalap, with frequently a small quantity of Ipecac combined with either of these, and followed by the common Tartar Emetic solution, in Elm-water, till the more violent symptoms were reduced. But I often found cases, where the patient was so violently taken, or where I had not been called till so late in the disease, that before the patient's system could be prepared, either by general or local depletion or mild aperients, followed by the judicious use of Tartar Emetic, during the height of each paroxysm, that for the administration of Quinine the golden moment had forever flown. These cases I also found were quite frequent. Again I have pushed the old treatment with every possible alacrity, caution, and judgment, of which I was capable, for days, without any abatement; when I at once resumed—taking advantage of the remissions—the use of large doses of Quinine, and with the immediate alleviation of the symptoms: and from that moment my patient would commence rapidly to recover. I also noticed the fact, that patients afflicted with Lung Fever, and treated with Quinine, premising other proper treatment, always recovered more rapidly, than under any other mode of treatment I had ever used. I also found the most marked benefit from its use, even where the redness of the tongue, burning of the stomach, with great thirst, and excessive purgation, seemed to forbid its use. In fact, I have given it, combined with Morphine, when the patient complained of extreme tenderness in the region of the stomach and bowels, retching to vomit; red, dry, and coated tongue, great thirst, and frequently excessive purging therewith, with the happiest effect in Pneumonitis. Yet, as a general rule, I would advise caution in its use, when such irritation of the stomach exists as a complication with Pneumoni ; and especially in the earlier stages of the disease. Still, in the latter stages, carefully observing the effect of it on the system, I have no hesitation in recommending its use, properly combined.

The result in the above cases, led me to try the virtues of this all-powerful medicine, in the earlier stages of Lung Fever, and before the system had time to become prepared as usual, for its reception; consequently I combined it with Mercury, Ipecac, and frequently Morphine. Sometimes using Dover's Powders instead of Ipecac and Morphine, where the patient seemed in the greatest danger, and with the effect of immediate relief. Often I would use the above combination during the intermision, or remission, and leave off as the hour for the rising of the fever had arrived, and have recourse to the Tartar Emetic in Elm-water, leaving off this whenever a remision took place again. In this way alternating, for a few days, assisted by local depletion, by means of cups over the inflamed part, I have frequently succeeded in breaking up the very worst forms of Lung Fever. But as Tartar Emetic seemed to possess greater influence over the different forms of Pneumonitis, than Ipecac, I was naturally led to try its efficacy, combined with Quinine, and giving at the same a sufficiency of Morphine to prevent its action on the bowels. Accordingly I settled down on the following prescription, varied according to circumstances.

When called to a case of Pneumonia in its earlier stages, say on the second or third day after; if the excitement were high, pain severe, and great difficulty in breathing; dry hacking cough; pulse 80 to 95, and often 100, full, and tolerably hard, or incompressible; tongue dry, and coated with a yellowish or brown fur, red at the tip and edges, with regular exacerbations, I first took blood from the arm, if the age, sex, and constitution of the patient would admit; at least, I cupped him freely over the chest, or seat of pain, drawing more or less blood in that way, and gave him Calomel, 10 grs.: Rhubarb, 16 grs.; followed by oil in seven or eight hours, unless it operated sufficiently of itself. As soon as the purgative effect of this dose was over, and the proper intermission had arrived, I gave him Quinine, from 5 to 10 grs.; Tartar Emetic, from $\frac{1}{8}$ to $\frac{1}{4}$ grs.; Morphine, $\frac{1}{8}$ to $\frac{1}{4}$ grs.; adding or leaving out the latter, as the nature of the case required, only giving a sufficiency to keep him calm, and prevent the Tartar from running off by his bowels; and I repeated this quantity every three hours for a greater or less time, stopping them should I think too high excitement had come up, or continuing them if I found they were cooling the fever, easing the pain, loosening the cough, and exciting a proper quantity of good billious stools, as they not unfrequently did. If I was compelled to stop them the first time, and again resort to local, or even general blood-letting, as was sometimes, though not often, the case, I again resumed their use, as soon as the next intermission took place. I seldom had to desist a second time, unless I did so merely to give the patient some little rest from medicine. I treated some twelve cases with local depletion, mild aperients, and then commenced the use of the above named medicine, so soon as there was a remission after the operation of the aperient. I soon began to prefer this mode of treatment to all others; and had recourse also to the use of Mercury (Calomel or Blue Pill,) combined therewith. Out of the twelve cases I treated in this way, not one died, and but one went over five days, without having all traces of the fever to disappear. More than one half of the cases, so far as my experience extends, are broken up by the fifth day, and the patients not in any degree weakened, as they generally were under the old treatment. I was frequently foiled in my efforts to cut short these cases, by the administration of Quinine by itself, and at one time even thought Quinine a dangerous agent in Pneumonia. This arose from the fact, that I did not understand giving it properly in this disease. In this part of Illinois, and especiallly on the Embarrass River, from its mouth up twenty-five miles, and especially for fifteen or twenty miles on either side, it is wonderfully sickly. Lying between this river and the Wabash, extending from the mouth, where it empties into the Wabash, for ten or twelve miles up the latter stream, and fifteen or twenty miles up the Embarrass, is a low, sandy tract of land, having a rich soil, most of which is subject to frequent overflows, and on, or near which, a wonderful degree of Intermittent and Remittent Fevers prevails, from May till November, and a peculiar form of Pneumonia from February till May, and all of which diseases must be cured principally by Mercury, Tartar Emetic, and Quinine. In no section of Illinois, not even in the American Bottoms, does that peculiar form of fevers, termed Malarious, prevail to a greater extent, than in this part of the Wabash Valley, and especially on the Embarras River.

Most parts of our county, (Lawrence) are quite healthy; but that portion lying immediately on the Embarras River, is extremely unhealthy. Indeed, all Illinois is subject to a large share of malarious disease. Our Winter Fevers partake more or less of that peculiar form of disease, and as Quinine in the Autumnal Intermittent and Remittent Fevers, is '*the medicine*,' so is it '*the medicine*' in our Winter Fevers, attended though they may be, and kept up by local inflammation. I have seen cases that have resisted the ordinary means of cure for days, and the patients had given up all hope of recovery, with one lobe of his lungs perfectly consolidated, yielding a dull, flat sound on percussion, and the vesicular murmur entirely absent, either anteriorly or posteriorly; with great difficulty of breathing, a feeble, quick pulse, cadaverous countenance, suddenly snatched from the grave by the following treatment:

℞ Quiniue, grs. ij; Tartar Emetic, $\frac{1}{8}$; Cayenne Pepper, $\frac{1}{2}$; Blue Mass, grs. 10, (and sometimes Calomel 5 to 8 grs. instead of the Mass,) and Sul. Morphine, $\frac{1}{4}$ grs., combined and made into one dose, and the like quantity, only leaving out the Morphine every time but the third one, to be given every three hours. Should the occasion demand it, I would also give an occasional dose of Syrup Squills, to enable the loose mucus to be freely thrown up. In addition to this treatment, I generally apply a large blister over the chest, and also give oil, or use injections, to keep up a sufficient action of the bowels; but am careful to guard against unnecessary purgation.—Under this mode of treatment, I have almost invariably seen them commence mending, and soon be up and about their business.

I have, like Dr. Stokes, always noticed Tartar Emetic exerts its most salutary effect on the human system, in the cure of inflammatory conditions of any organ, when it neither vomits nor purges. Combined with Quinine, it does not prostrate the nervous system as it does uncombined, while the Quinine is prevented from acting to such an extent as a stimulant, as when uncombined with Tartar: while its sedative and febrifuge properties are increased by the union. The Morphine also prevents the Tartar from running off by the bowels, and gives it a chance to exert its powerful properties on the system. The Mercury is a most valuable combination in the latter stages, and necessarily precedes the above in the earlier stages. Every practitioner in the Mississippi Valley, must have felt the dangerous position of being called to treat a bad case of Pneumonia, and finding his ordinary means fail, has resorted to Quinine, as directed by some of our best authorithorities, and found it fail also. But my word for it, in nine cases out cf ten, where these failures are experienced, if resort was had to Quinine, Tartar Emetic, and Morphine, also Mercury, if the state of the secretions demanded it, combined in different proportions, according to circumstances, immediate relief would be the result, and by perseverance, a complete cure effected.

Many have been induced, from reading the works of our systematic writers of the day, to try the benefits of Quinine in Pneumonia, and other local inflammations combined with a malignant form of fever, and have not met with that success they were induced to believe would be the result, and hence have abandoned it in perfect disgust. But if they will make the combination I have here laid down, as near as the circumstances of each individual case will permit, using as adjuvants, local depletion, and mild aperients, as often as required, they will be most happily disappointed.

As I remarked in the outset, it sometimes becomes necessary to bleed generally and freely, by opening a vein in the arm, and following that with a brisk cathartic and the use of cups to the part, frequently applied and then the above combination will exert its most salutary influence on the system. I seldom use Rhubarb in the way Dr. Howland has advised, nor the Soda, and hence cannot speak as to their beneficial qualities in such combination, though I have no doubt but that they often operate kindly. I use mucilaginous drinks from the commencement, in Pneumonia. I have seen, and indeed it is common with me, to have Pneumonia disappear in the course of from three to five days; whereas I used to think myself well off if I cured a case in from nine to fourteen days.—Patients treated in this way, get right up whenever the fever leaves them, and regain their strength very rapidly; while those that are treated in the old fashioned way, get out of bed slowly, regain their strength slowly, and are much more liable to relapse, than when treated by Quinine, &c.

Another grand reason why Tartar Emetic ought to be combined with Quinine, Morphine, &c., is because you can give the Quinine with the greatest safety and most beneficial results at the commencement of the attack, only waiting, as a general thing, to reduce the excitement a little, by general or local depletion; to open the bowels by means of a mild but brisk purgative, which will not take, usually, over five hours; when you commence giving the great anti-periodic and febrifuge medicine, combined with the most powerful sedative and anodyne agents; and when Calomel or Blue mass be given also, you have at once, and in one combination, the medicines that combine the most powerful curative properties; and which would, so combined, in this miasmatic region, come nearer being a cure-all, or Panacea, than any other article of medi- within the knowledge of man.

